# Fear of falling as a mediator in the association between social frailty and health-related quality of life in community-dwelling older adults

**DOI:** 10.1186/s12877-023-04144-1

**Published:** 2023-07-10

**Authors:** Kuan-Ying Wu, Duan-Rung Chen, Chang-Chuan Chan, Yen-Po Yeh, Hsiu-Hsi Chen

**Affiliations:** 1 Institute of Health Policy and Management, College of Public Health, National Taiwan University, Taipei, Taiwan; 2 Innovation and Policy Center for Population Health and Sustainable Environment, College of Public Health, National Taiwan University, Taipei, Taiwan; 3 Institute of Health Behaviors and Community Sciences, College of Public Health, National Taiwan University, Taipei, Taiwan; 4 Population Health Research Center, College of Public Health, National Taiwan University, Taipei, Taiwan; 5 Institute of Environmental and Occupational Health Science, College of Public Health, National Taiwan University, Taipei, Taiwan; 6 Changhua County Public Health Bureau, Changhua County, Taiwan; 7 Graduate Institute of Epidemiology and Preventive Medicine, College of Public Health, National Taiwan University, Taipei, Taiwan

**Keywords:** Social frailty, Fear of falling, Health-related quality of life, Community-dwelling older adults

## Abstract

**Background:**

Social frailty is associated with Fear of Falling (FoF) and health-related quality of life (HrQoL). However, how social frailty simultaneously influences FoF and HrQoL remains unclear. The study aims to understand the links between social frailty, FoF, and HrQoL in older adults and the mediating role of FoF in the relations between social frailty and HrQoL.

**Methods:**

In this cross-sectional survey, 1,933 community-dwelling older adults from Changhua County, Taiwan, were interviewed using a self-administrated questionnaire. In total, 1,251 participants with complete data were included for analysis. Data were analyzed using the SPSS PROCESS macro. A simple mediation was employed using social frailty as the independent variable, FoF as the mediator variable, and HrQoL as the outcome variable.

**Results:**

Social frailty was associated with HrQoL and indirectly with HrQoL through FoF, and FoF was directly associated with HrQoL. Of the 5-item social frailty index, “going out less frequently” was correlated with HrQoL and indirectly with HrQoL through FoF. Individuals who felt unhelpful toward family or friends had the worst physical HrQoL and did not talk to someone daily had the most negative influence on mental HrQoL.

**Conclusions:**

Social frailty can directly and indirectly, through FoF decrease HrQoL. It also emphasizes the importance of social connectivity in reducing the risk of falls. This study points to the need for social connectivity and fall prevention programs as essential components of strategies to enhance the health and well-being of community-dwelling older adults.

**Supplementary Information:**

The online version contains supplementary material available at 10.1186/s12877-023-04144-1.

## Introduction

Health-related quality of life (HrQoL) plays a vital role in the health of older adults. A better understanding of the determinants of HrQoL is increasingly necessary for an aging population [1, 2]. HrQoL is a multidimensional construct measuring the subjective appraisal of health status through daily physical, mental, and social functioning [1–3]. Effective intervention to improve HrQoL has been a realistic goal of many health systems instead of merely focusing on survival [1, 2].

Frailty is a concept that encompasses physical, psychological, and social vulnerability [4, 5]. Previous studies have focused on the physical frailty of older adults [6–8]. However, in an aging society, older adults often face various social problems, such as changes in family structure, economic status, and social participation [9]. Gobbens et al. were the first to propose the concept of social frailty, a decline in social relations, social support, and living alone [10]. Bunt et al. indicated social frailty as a multifaceted concept, a continuum of being at risk of losing or losing social resources and lacking social behaviors, social activities, and self-management abilities to fulfill basic social needs [11]. A study explores the dimensions of social vulnerability from a social ecology perspective, demonstrating that social support, engagement in social activities, relations with others, living situation, self-esteem, sense of control, and contextual socioeconomic status were the seven emergent factors from the Principal Component Analysis [12]. Previous studies used social frailty and social vulnerability as synonyms [13]. Van Oostrom et al. operationalize social frailty as loneliness, low social support, and limited social participation [14]. A systematic review indicated that measures of frailty’s social dimension varied among different instruments. The most frequently used component covered social support, social activities, social network, loneliness, and living alone [15]. In summary, social frailty was designed to show an overall situation indicating the disadvantages of an individual’s social existence or circumstances.

Studies indicated that social frailty could predict physical frailty, disability, and mortality among community-dwelling older adults [9, 16]. Moreover, in a previous study, when both physical and psychological frailty were controlled for, a significant relationship was observed between social frailty and physical and mental HrQoL [2]. Therefore, social frailty is essential when exploring older adults’ health outcomes and well-being.

Apart from the quality of life in older adults, falls are pernicious to the health of older adults [1], and almost one in every three community-dwelling older adults experience a fall within a year [17]. Poor health consequences of falls include bodily injuries, disability, and HrQoL decline [18, 19]. In addition to falls, fall-related risks, such as Fear of Falling (FoF), can negatively affect the HrQoL of older adults [1, 20, 21]. FoF is an attitude of caution toward falling [22]. Among individuals aged 65 and above, the prevalence rate of FoF is approximately 23% to 85% [21, 23–27]. Even among community-dwelling older adults who have not experienced falls, about 66% report FoF [25]. Therefore, FoF is considered an independent predictor of HrQoL regardless of whether the individual had fallen before [28].

FoF can reduce physical and mental performance, increase the risk of falling, and restrict participation in activities and social networks [21, 27, 29]. Older adults experiencing FoF tend to reduce social interaction, lowering their quality of life and well-being [30] and decreasing the HrQoL [1, 20, 21, 31]. However, family, friends, or community support can enhance an individual’s confidence to manage their fear of falling [32, 33]. The cross-sectional data analysis results indicated that social frailty was correlated with FoF, and the longitudinal data analysis demonstrated that social frailty could significantly predict perceived fear of falls [34]. However, the association between social support or social interaction and FoF is inconclusive, and how social frailty simultaneously affects FoF and HrQoL remains to be seen. As previous studies independently explored the relationship between social frailty and HrQoL [2, 35–37] and between FOF and HrQoL [1, 20, 21, 31], this study aims to help clarify how these factors interact and influence each other, offering a more comprehensive understanding of the complex interplay between them. This study aims to extend the understanding of the impact of social frailty on HrQoL by illuminating the mediating role of FOF. Doing so can provide a new framework for considering and addressing social frailty and fear of falling simultaneously to improve HrQoL among older adults.

**Fig. 1:**
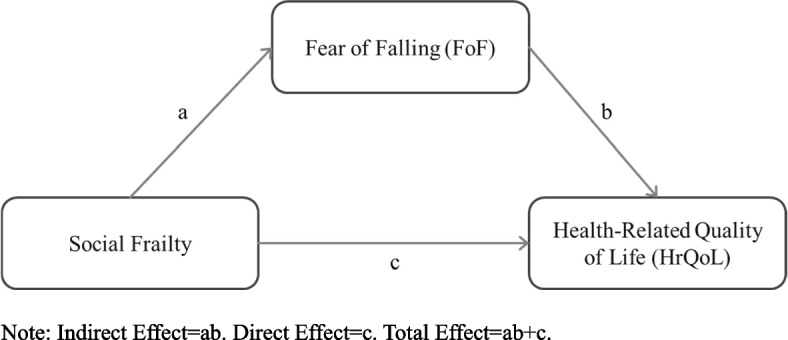
DAG shows the medication model of the relationships among social frailty, FoF, and HrQoL

The study aims to understand the mediating role of FoF in the relations between social frailty and HrQoL. We hypothesize that 1) social frailty may affect HrQoL (the direct effect of “c” in Fig. 1) and FoF (the direct effect of “a”); 2) FoF may affect HrQoL (the direct effect of “b”); 3) social frailty also affects HrQoL through FoF (the indirect effect of “ab” in Fig. 1). We further examine each aspect of social frailty to highlight the essential factor associated with HrQoL. The purpose is to highlight the most notable index for policymakers and public health practitioners that may be helpful in efforts to reduce social frailty status among older adults.

## Methods

### Study participants

It is a cross-sectional survey, and 1,933 community-dwelling older adults were recruited from community care centers in Changhua County, Taiwan, under a large-scale community-integrated health screening project implemented by Changhua County Health Bureau. The integrated health project provides comprehensive health screenings for citizens aged 30 and above, including blood serum tests cancer, and chronic disease. A questionnaire regarding frailty was collected through face-to-face interviews with community-dwelling older adults from October 2019 to August 2020. Public health practitioners or medical professionals conducted the interviews. The inclusion criteria were as follows: (1) being aged 65 or above, (2) being a community-dwelling older adult capable of self-care, and (3) being able to communicate in Mandarin or Taiwanese. Individuals with cognitive impairments, mental disorders, or severe disabilities and individuals living in long-term care facilities were excluded. All participants were informed of the study’s objectives, and informed consent was obtained before each interview (IRB No: 201903HS026).

### Measurements

#### Health-related quality of life

We used the 8-item Short-Form Health Survey scale (SF-8) to assess physical and mental HrQoL. The SF-8, a short version of the original 36-item scale (SF-36), is widely used to determine HrQoL [38]. The SF-8 comprises eight items that assess general health perceptions, physical functioning, role limitations, and the degree of bodily pain resulting from physical health problems. It is also used to determine mental health-related issues, including vitality, social functioning, and limitations in functioning resulting from emotional problems [6, 39]. The SF-8 has a weighted Physical Component Summary (PCS) and Mental Component Summary (MCS) score by calculating the scores of each item and using norm-based scoring methods. The means (T-scores) are 50, and the standard deviation is 10; means, variances, and regression weights are normalized to the general US population as a reference [6, 39]. The scores range from 0 to 100 (lowest to highest level of health). The higher scores reveal better health [6, 39]. PCS and MCS can reflect the older adult respondent’s physical and mental health status. Previous studies using the Chinese and Spanish versions of the SF-8 scale have demonstrated that Cronbach’s alpha was 0.85 and 0.92, respectively [38, 40].

#### Social frailty

We used a 5-item social frailty index [41]: (1) “going out less frequently compared with last year,” (2) “rarely visiting friends,” (3) “feeling unhelpful toward family or friends,” (4) “living alone,” and (5) “talking with someone every day.” Answers with “Yes” to Questions 1, 3, and 4 and “No” to Questions 2 and 5 were considered negative responses. A total score of 0, 1, and 2–5 indicated that the respondent was “healthy,” “prefrail,” and “frail,” respectively [16, 41–43]. The validity of the social frailty index was supported by previous studies [16, 41–43].

#### Fear of falling

The Falls Efficacy Scale International (FES-I) comprises 16 items that assess the level of concern toward falling while performing daily activities. These activities include dressing, bathing, sitting down or standing up from a chair, climbing up or down the stairs, reaching up or bending down, walking up or down slopes, and participating in social activities [44]. The short version of the FES-I comprises seven items from the original version (items 2, 4, 6, 7, 9, 15, and 16). According to Kuo et al., both versions’ responses are strongly correlated (Spearman’s ρ = 0.963) [6]. In addition, the short version of the FES-I is negatively associated with PCS and MCS, independent of physical frailty [6]. Therefore, in the analyses, we included the scores obtained with the short version of the FES-I, graded on a 4-point Likert scale. Higher scores indicate more significant concern toward falling. The Cronbach’s alpha for the Chinese version short FES-I was reported as about 0.92 [6].

#### Covariates

The variables on the respondent’s characteristics included sex, age, marital status, educational attainment, physical frailty, disability, comorbidity, and experience of falls during the preceding year. We coded the categorical variables as follows: (1) sex: 1 for male and 2 for female; (2) marital status: 1 for married and 2 for divorced/widowed/single; (3) educational attainment: 0 for non-educated, 1 for primary school, 2 for junior high school, 3 for senior/vocational high school, and 4 for college and above; (4) disability status: 1 for having disability officially confirmed by the government (individuals will be assessed physical, mental, cognitive, and social status by medical professionals using the Functioning Disability Evaluation Scale (FUNDES)), and 0 for not having. This scale is based on the standard in the International Classification of Functioning, Disability, and Health (ICF) [45].); (5) comorbidity: 1 for having comorbidity and 0 for not having comorbidity; and (6) experience of falls during the preceding year: 1 for yes and 0 for no. We used five items to assess physical frailty: three from the Study of Osteoporotic Fractures (SOF) index and two on-site physical measurement items. The five items were based on the following: (1) the question “Have you lost more than 5% of your weight unintentionally last year?” (2) the question “Do you often feel exhaustion or poor endurance while doing things over the last week (more than 3 days in a week)?,” (3) the ability to stand up from a chair five times without using one’s hand for support, (4) hand grip strength, and (5) 6-m straight walking time. These five items are consistent with the conceptualization of Fried’s frailty phenotype [46].

### Statistical analysis

Categorical variables are frequencies and percentages, and continuous variables are represented as means and standard deviations (SDs). Spearman’s rank correlation was used to test the relation between the various variables. Mediation analysis was performed using SPSS PROCESS macro version 4.0 with Model 4, a simple mediation model [47].

Figure 1 shows the theoretical framework for social frailty, FoF, and HrQoL relationships. For HrQoL, PCS and MCS were the separate outcome variables, and FoF was the mediator variable. We further examine the relationship between each indicator of social frailty and FoF and HrQoL, as indicated in Fig. 1. Five indicators of “social frailty” include 1) going out less frequently; 2) rarely visiting friends; 3)feeling unhelpful toward family or friends; 4) not talking to someone every day, and 5) living alone.

All the participants’ demographic characteristics, including sex, age, marital status, educational attainment, physical frailty, disability, comorbidity, and falls experiences during the preceding year, were controlled for all the mediation models. Bootstrapping (5,000 resamples) was used to estimate the 95% confidence interval (CI) for the abovementioned effects. All statistical analyses were performed using IBM SPSS Statistics version 22 (IBM, Armonk, NY, USA).

## Results

### Descriptive statistics

A total of 1,251 community-dwelling older adults were included in the analysis after excluding participants with missing data. Among these participants, in terms of social frailty, 545 (43.6%) were categorized as non-frail, 434 (34.7%) were classified as prefrail, and 272 (21.7%) were categorized as frail. The primary characteristics of the study participants are presented in Table 1. The mean age of the study participants was 76.5 years (SD = 6.9), with the majority being women (77.6%). About 50.8% of the participants were divorced, widowed, or single; 43.4% were illiterate; 55.8% were categorized as prefrail in terms of physical frailty; 83.9% were living with others; 93.1% were not disabled; 79.8% did not have any comorbidities, and 79.0% had no history of falls during the preceding year.

**Table 1:** Primary characteristics of the study participants, Mean ± SD or n (%)

Variable	Overall (*n* = 1,251)	Social Frailty			*P*-value*
		Nonfrail (*n* = 545)	Prefrail (*n* = 434)	Frail (*n* = 272)	
Gender, n (%)					0.816
Female	971 (100)	421 (43.4)	335 (34.5)	215 (22.1)	
Male	280 (100)	124 (44.3)	99 (35.4)	57 (20.4)	
Age, Mean ± SD (years)	76.5 ± 6.9	75.5 ± 6.8	76.4 ± 6.8	78.9 ± 6.5	< 0.001
Marital Status, n (%)					< 0.001
Married	616 (100)	314 (51)	204 (33.1)	98 (15.9)	
Others	635 (100)	231 (36.4)	230 (36.2)	174 (27.4)	
Education level, n (%)					0.010
Non-educated	543 (100)	222 (40.9)	178 (32.8)	143 (26.3)	
Primary School	497 (100)	221 (44.5)	181 (36.4)	95 (19.1)	
Junior High School	95 (100)	45 (47.4)	37 (38.9)	13 (13.7)	
Senior/vocational High School	78 (100)	34 (43.6)	25 (32.1)	19 (24.4)	
College and above	38 (100)	23 (60.5)	13 (34.2)	2 (5.3)	
Physical Frailty, n (%)					< 0.001
Nonfrail	464 (100)	254 (54.7)	152 (32.8)	58 (12.5)	
Prefrail	698 (100)	274 (39.3)	251 (36)	173 (24.8)	
Frail	89 (100)	17 (19.1)	31 (34.8)	41 (46.1)	
Living Situation, n (%)					< 0.001
Living with others	1,050 (100)	545 (51.9)	337 (32.1)	168 (16)	
Living alone	201 (100)	0 (0)	97 (48.3)	104 (51.7)	
Disability, n (%)					0.662
No	1,165 (100)	510 (43.8)	405 (34.8)	250 (21.5)	
Yes	86 (100)	35 (40.7)	29 (33.7)	22 (25.6)	
Morbidity, n (%)					0.056
No	253 (100)	127 (50.2)	76 (30)	50 (19.8)	
Yes	998 (100)	418 (41.9)	358 (35.9)	222 (22.2)	
Fall History during the Last Year, n (%)					< 0.001
No	988 (100)	451 (45.6)	345 (34.9)	192 (19.4)	
Yes	263 (100)	94 (35.7)	89 (33.8)	80 (30.4)	

*SD* Standard deviation

^*^Chi-Squared Test for proportions and One-way ANOVA test for continuous measures

### Correlations among variables

Table 2 presents the correlations among all variables. Those who were “social frailty,” “going out less frequently,” “rarely visiting friends,” “feeling unhelpful toward family or friends,” “not talking to someone every day,” “living alone,” and FoF was negatively related to PCS, also in MCS. In addition, those who were “social frailty,” “going out less frequently,” “rarely visiting friends,” “feeling unhelpful toward family or friends,” and “living alone” was positively correlated with FoF.

**Table 2:** Correlations between the study variables for PCS and MCS (*N* = 1,251)

Variables	1	2	3	4	5	6	7	8
**PCS**								
1. Social Frailty	1							
2. Going out less frequently	.574**	1						
3. Rarely visiting friends	.603**	.071*	1					
4. Feeling unhelpful toward family or friends	.496**	.072*	.236**	1				
5. Not talking to someone every day	.315**	.019	.219**	.195**	1			
6. Living alone	.421**	-.029	.019	.067*	.036	1		
7. FOF	.208**	.136**	.123**	.123**	.008	.086**	1	
8. PCS	-.270**	-.183**	-.151**	-.193**	-.105**	-.081**	-.328**	1
**MCS**								
1. Social Frailty	1							
2. Going out less frequently	.574**	1						
3. Rarely visiting friends	.603**	.071*	1					
4. Feeling unhelpful toward family or friends	.496**	.072*	.236**	1				
5. Not talking to someone every day	.315**	.019	.219**	.195**	1			
6. Living alone	.421**	-.029	.019	.067*	.036	1		
7. FOF	.208**	.136**	.123**	.123**	.008	.086**	1	
8. MCS	-.250**	-.171**	-.144**	-.125**	-.104**	-.098**	-.266**	1

*FOF* Fear of falling, *PCS* Physical Component Summary, *MCS* Mental Component Summary

^*^p < .05, ** *p* < .01

### Mediation analysis

We performed a simple mediation analysis using the SPSS PROCESS macro, Model 4. The covariates were controlled for each of the following mediation models. The results are presented in Table 3.

**Table 3:** Bootstrap results of social frailty indices through FoF (mediator) on PCS and MCS

Domain/Effect	PCS			MCS		
	*B*	*95% CI*		*B*	*95% CI*	
***Social Frailty***						
Direct	-1.65	-2.09	-1.21	-1.18	-1.53	-0.83
Indirect	-0.28	-0.45	-0.13	-0.15	-0.27	-0.07
Total	-1.93	-2.39	-1.47	-1.33	-1.69	-0.97
***Going out less frequently***						
Direct	-1.87	-2.76	-0.99	-1.44	-2.14	-0.74
Indirect	-0.46	-0.81	-0.17	-0.25	-0.48	-0.08
Total	-2.34	-3.25	-1.42	-1.69	-2.40	-0.99
***Rarely visiting friends***						
Direct	-2.02	-2.97	-1.07	-1.38	-2.13	-0.63
Indirect	-0.31	-0.64	-0.01	-0.17	-0.37	0.00
Total	-2.33	-3.32	-1.34	-1.55	-2.31	-0.78
***Feeling unhelpful toward family or friends***						
Direct	-2.94	-4.18	-1.71	-0.94	-1.92	0.04
Indirect	-0.42	-0.87	0.00	-0.24	-0.51	0.00
Total	-3.36	-4.65	-2.08	-1.17	-2.18	-0.17
***Not talking to someone every day***						
Direct	-2.99	-4.96	-1.03	-3.20	-4.74	-1.65
Indirect	0.11	-0.67	0.87	0.06	-0.37	0.50
Total	-2.88	-4.94	-0.82	-3.13	-4.72	-1.55
***Living alone***						
Direct	-1.06	-2.21	0.09	-1.31	-2.22	-0.40
Indirect	-0.39	-0.83	-0.01	-0.21	-0.47	0.00
Total	-1.45	-2.66	-0.25	-1.52	-2.45	-0.60

Bootstrap resample size = 5,000

All covariates are controlled in all of the equations for the mediation analyses

*B* Unstandardized regression coefficients, *CI* 95% Confidence Interval, *PCS* Physical Component Summary, *MCS* Mental Component Summary, *Indirect effect* Through fear of falling

When FoF served as a mediating variable, social frailty showed a negative relationship with PCS and indirectly with PCS. The total effect of the regression coefficient (*B*) of “social frailty” on PCS was −1.93. Of the 5-item social frailty index, individuals who went out less frequently, rarely visited friends, felt unhelpful toward family or friends, did not talk to someone daily, and lived alone showed a significant negative relationship with PCS. Those who went out less frequently, rarely visited friends, and lived alone, showed an indirectly negative relationship with PCS through FoF. In addition, the index of “feeling unhelpful toward family or friends” had the most significant effect on PCS (*B* =  −3.36).

Regarding the factors associated with MCS, social frailty is negatively associated with MCS and indirectly with MCS through FoF. The total effect of the regression coefficient (*B*) of “social frailty” on MCS was −1.33. In each social frailty index, individuals who went out less frequently, rarely visited friends, did not talk to someone daily, and lived alone showed a significantly negative relationship with MCS. Those who went out less frequently showed a significantly indirectly negative relationship with MCS through FoF. The “not talking to someone every day” index significantly affected MCS (*B* =  −3.13).

The details about the associations of “a,” “b,” and “ab” in Fig. 1 from the mediation models are shown in Supplementary Table 1 in Additional file 1.

Covariates such as “physical frailty” and “morbidity” had a significant effect on PCS, and “physical frailty,” “disability,” and “fall history during the last year” have a considerable impact on MCS in all mediation models (see Additional file 1: Supplementary Table 2).

## Discussion

### Main findings

We examined the relationship between social frailty (using the 5-item social frailty index) and HrQoL with FoF as a mediator. The results indicated that social frailty was negatively associated with PCS and MCS when FoF was a mediating variable and indirectly with PCS and MCS through FoF. “Going out less frequently” in the 5-item social frailty index showed a significantly negative correlation with PCS and MCS and indirectly with PCS and MCS through FoF. In addition, “feeling unhelpful toward family or friends” had the most considerable effect on PCS, and “not talking to someone every day” had the most notable effect on MCS.

### Comparison with previous findings

Several researchers have highlighted that the lack of social contact, relationships, and support affects the quality of life [2, 35–37] and that social frailty is related to physical and mental HrQoL, even when physical and psychological frailty are accounted for [2]. In this study, social frailty was directly associated with HrQoL and indirectly with HrQoL through FoF, and FoF was negatively associated with HrQoL.

Social frailty is significantly associated with FoF, suggesting that older adults who were socially inactive may experience a sense of FoF. Additionally, the FoF was associated with physical and mental HrQoL when physical frailty and other covariates were controlled for [6]. Previous studies have demonstrated the associations between social frailty and HrQoL [2, 35–37] and between FoF and HrQoL [1, 20, 21, 31]. Still, they did not clarify how social factors influence HrQoL when FoF acts as a mediator. This study demonstrated that the lack of social interactions might reduce HrQoL and that FoF partially mediates the relationship between social frailty and HrQoL.

Among the participants of this study, 35.3% were social prefrailty, and 21.4% were social frailty. In other words, more than half of the participants met at least one or more social frailty indices. Changhua County is located in Midwest Taiwan and ranks third among Taiwanese counties in the value of its agriculture, forestry, fishery, and animal husbandry output [48]. Nearly 80% of the participants were from rural communities in Changhua County, among whom 35.2% were social prefrailty, and 21.9% were social frailty. Among those from urban communities, 35.7% were social prefrailty, and 19.9% were social frailty. These differences in the prevalence rate of social frailty between rural and urban communities were not statistically significant in the present study sample. For rural areas in other countries, the prevalence rate of social frailty is approximately 20.5% in the Pyeongchang rural area in Korea [42], 8.9% in the rural villages of Spain [49], and 4.1% in Doetinchem in the Netherlands [14]. For urban areas in other countries, the prevalence rate of social frailty is 18.0% in Shiga Prefecture in Japan and 18.4% in Singapore [9, 50]. Overall, the percentage of social frailty varies across studies in different countries or areas, which may be because of differences in the social frailty scales used and the ages of participants.

There is no standard gold measurement for social frailty [15]. Previous studies considered social frailty as rarely interacting with others, lack of social support, social activities, social networks, and loneliness or living alone [9, 15]. This study used the 5-item social frailty index [41], including going out less frequently, rarely visiting friends, feeling unhelpful toward family or friends, not talking to someone daily, and living alone to demonstrate that overall poor and declined social conditions that might result in adverse health outcomes.

Our findings also revealed that the “physical frailty” covariate strongly predicts PCS and MCS. Studies have also indicated a strong correlation between physical frailty and physical HrQoL [2, 7]. However, in a longitudinal study, social frailty can affect physical frailty for individuals who were not physically frail or prefrail at baseline during a 4-year follow-up [16]. Therefore, preventing community-dwelling older adults from experiencing social and physical frailty is essential to improving their HrQoL.

### Policy implications

This study demonstrated that social frailty or the lack of social connections, relationships, or contact affects the HrQoL of community-dwelling older adults. Generally, social interaction is beneficial for psychological and health outcomes, and social contacts can make people happy even in interaction with strangers or acquaintances [51]. Therefore, increasing Vitamin S (Vitamin Social Contact) may enhance community-dwelling older adults’ happiness and well-being [51].

This study found that improvements in social connections (even through interactions with strangers) can reduce the risk of falling. A systematic review highlighted FoF’s pernicious effects, such as decreased quality of life, reduced social contact, and physical activity, increased declining incidence, and increased depression [21]. Another study indicated that approximately 41.7% of those who reported FoF at baseline experienced at least one fall 2 years later [26]. Falling has a considerable social and economic burden; therefore, enhancing social support and interactions to prevent older adults from falling is a public health challenge [32, 33, 52].

This study indicated that social frailty plays a significant role in the HrQoL of older adults and that this influence is mediated, at least in part, by FoF. Socially frail older adults may fear falling and restrict their activities, which could further diminish their HrQoL. The significant role of FoF in reducing HrQoL underscores the importance of developing and implementing fall-prevention programs as part of public health strategies for older adults.

This study makes several contributions to gerontology literature, particularly in understanding the links between social frailty, FoF, and HrQoL in older adults. One of the key findings of the study is the mediation role of FoF. We found that social frailty impacts HrQoL directly and indirectly through FoF. This adds a new dimension to our understanding of the social factors affecting the health of older adults. The study also dissects the different elements of the social frailty index, identifying which aspects have the most significant impact on physical and mental HrQoL. It highlights the impact of specific social behaviors, like going out less frequently or not talking to someone daily, on older adults’ HrQoL. The results highlight the need for interventions to improve social connectivity and fall prevention in older adults. This may encourage policy-makers to focus on these aspects, potentially leading to more effective programs and interventions to enhance the health and quality of life of older adults.

### Limitations

This study has some limitations. First, the study participants were recruited only from Changhua County, limiting the results’ generalizability. Second, the data obtained from the questionnaire were self-reported, which may have resulted in recall bias. Third, the study had a cross-sectional design, which precluded inference of the causal relationship between social frailty, FoF, and HrQoL, which can be addressed in future longitudinal studies.

## Conclusion

Social frailty can decrease the extent of HrQoL and indirectly reduce HrQoL through FoF. Preventing social frailty, improving social connections, and developing fall-prevention programs can help enhance community-dwelling older adults’ health and well-being.

## Supplementary Information

**Additional file 1: Sup_Table 1.** Mediation Analyses: Unstandardized Regression Coefficients. **Sup_Table 2.** Mediation Analyses: Unstandardized Regression Coefficients of Covariates.

## Data Availability

Data is available upon request. Please get in touch with correspondent author Duan-Rung Chen (e-mail: duan@ntu.edu.tw).

## Abbreviations

FoF: Fear of falling
HrQoL: Health-related Quality of Life
SF-8: 8-Item Short-Form Health Survey
SF-36: 36-Item Short Form Health Survey
PCS: Physical Component Summary
MCS: Mental Component Summary
FES-I: The Falls Efficacy Scale International
SOF: Study of Osteoporotic Fractures
SDs: Standard deviations
CI: 95% Confidence interval
Vitamin S: Vitamin Social Contact
