# Supplementary material for: Fear of falling as a mediator in the association between social frailty and health-related quality of life in community-dwelling older adults
Source: BMC Geriatr. 2023 Jul 10;23:421. doi: 10.1186/s12877-023-04144-1 (PMC10334636; doi:10.1186/s12877-023-04144-1)
Supplement: Supplementary file 1 — Additional file 1: Sup_Table 1. Mediation Analyses: Unstandardized Regression Coefficients. Sup_Table 2. Mediation Analyses: Unstandardized Regression Coefficients of Covariates. [file 12877_2023_4144_MOESM1_ESM.pdf]

Sup_Table 1 Mediation Analyses: Unstandardized Regression Coefficients

Variable/Effect	*B*	*SE*	*t*	*p*	*95% CI*	
Social Frailty						
SF→PCS	-1.65	0.23	-7.29	＜0.001	-2.09	-1.21
SF→FOF	0.34	0.08	4.32	＜0.001	0.18	0.49
SF→FOF→PCS	-0.84	0.08	-10.20	＜0.001	-1.00	-0.68
SF→MCS	-1.18	0.18	-6.58	＜0.001	-1.53	-0.83
SF→FOF	0.34	0.08	4.32	＜0.001	0.18	0.49
SF→FOF→MCS	-0.45	0.06	-6.96	＜0.001	-0.58	-0.32
Going out less frequently						
Going out less frequently→PCS	-1.87	0.45	-4.16	＜0.001	-2.76	-0.99
Going out less frequently→FOF	0.53	0.15	3.45	＜0.001	0.23	0.83
Going out less frequently→FOF→PCS	-0.88	0.08	-10.56	＜0.001	-1.04	-0.71
Going out less frequently→MCS	-1.44	0.36	-4.06	＜0.001	-2.14	-0.74
Going out less frequently→FOF	0.53	0.15	3.45	＜0.001	0.23	0.83
Going out less frequently→FOF→MCS	-0.48	0.07	-7.31	＜0.001	-0.61	-0.35
Rarely visiting friends						
Rarely visiting friends→PCS	-2.02	0.48	-4.18	＜0.001	-2.97	-1.07
Rarely visiting friends→FOF	0.35	0.17	2.09	0.04	0.02	0.67
Rarely visiting friends→FOF→PCS	-0.89	0.08	-10.73	＜0.001	-1.05	-0.72
Rarely visiting friends→MCS	-1.38	0.38	-3.60	＜0.001	-2.13	-0.63
Rarely visiting friends→FOF	0.35	0.17	2.09	0.04	0.02	0.67
Rarely visiting friends→FOF→MCS	-0.49	0.07	-7.49	＜0.001	-0.62	-0.36
Feeling unhelpful toward family or friends						
Feeling unhelpful→PCS	-2.94	0.63	-4.67	＜0.001	-4.18	-1.71
Feeling unhelpful→FOF	0.48	0.22	2.20	0.03	0.05	0.90
Feeling unhelpful→FOF→PCS	-0.89	0.08	-10.72	＜0.001	-1.05	-0.72
Feeling unhelpful→MCS	-0.94	0.50	-1.88	＞0.05	-1.92	0.04
Feeling unhelpful→FOF	0.48	0.22	2.20	0.03	0.05	0.90
Feeling unhelpful→FOF→MCS	-0.50	0.07	-7.57	＜0.001	-0.62	-0.37
Not talking to someone every day						
Not talking to someone→PCS	-2.99	1.00	-2.99	＜0.001	-4.96	-1.03
Not talking to someone→FOF	-0.12	0.34	-0.36	＞0.05	-0.80	0.55
Not talking to someone→FOF→PCS	-0.91	0.08	-10.99	＜0.001	-1.07	-0.75
Not talking to someone→MCS	-3.20	0.79	-4.06	＜0.001	-4.74	-1.65
Not talking to someone→FOF	-0.12	0.34	-0.36	＞0.05	-0.80	0.55
Not talking to someone→FOF→MCS	-0.51	0.07	-7.77	＜0.001	-0.63	-0.38
Living alone						
Living alone→PCS	-1.06	0.59	-1.81	＞0.05	-2.21	0.09
Living alone→FOF	0.44	0.20	2.18	0.03	0.04	0.83
Living alone→FOF→PCS	-0.90	0.08	-10.82	＜0.001	-1.06	-0.74
Living alone→MCS	-1.31	0.46	-2.84	＜0.001	-2.22	-0.40
Living alone→FOF	0.44	0.20	2.18	0.03	0.04	0.83
Living alone→FOF→MCS	-0.49	0.07	-7.52	＜0.001	-0.62	-0.36

Note: B: Unstandardized regression coefficients. CI: 95% Confidence Interval

Bootstrap resample size=5,000

All covariates are controlled in all of the equations for the mediation analyses.

FOF: Fear of falling, PCS: Physical Component Summary, MCS: Mental Component Summary

Sup_Table 2 Mediation Analyses: Unstandardized Regression Coefficients of Covariates

Domain/Effect	PCS			MCS		
	B	*95% CI*		*B*	*95% CI*	
Social Frailty						
Gender	-0.41	-1.41	0.59	-0.33	-1.13	0.46
Age	-0.01	-0.07	0.06	0.06	0.01	0.12
Marital Status	-0.56	-1.41	0.29	-0.11	-0.78	0.56
Education level	0.39	-0.04	0.82	-0.17	-0.51	0.17
Physical Frailty	-0.86	-1.33	-0.39	-1.35	-1.73	-0.98
Disability	-1.18	-2.74	0.39	-1.37	-2.61	-0.13
Morbidity	-1.76	-2.74	-0.78	-0.33	-1.11	0.44
Fall History during the Last Year	-0.49	-1.46	0.49	-0.84	-1.61	-0.07
Going out less frequently						
Gender	-0.35	-1.37	0.66	-0.3	-1.10	0.51
Age	-0.02	-0.09	0.04	0.05	0.00	0.11
Marital Status	-0.96	-1.81	-0.1	-0.4	-1.07	0.28
Education level	0.39	-0.05	0.82	-0.17	-0.52	0.17
Physical Frailty	-1.06	-1.53	-0.59	-1.49	-1.86	-1.12
Disability	-1.26	-2.85	0.33	-1.43	-2.68	-0.18
Morbidity	-1.71	-2.70	-0.71	-0.29	-1.08	0.49
Fall History during the Last Year	-0.67	-1.66	0.32	-0.97	-1.76	-0.19
Rarely visiting friends						
Gender	-0.25	-1.27	0.76	-0.23	-1.04	0.57
Age	-0.02	-0.08	0.05	0.06	0.01	0.11
Marital Status	-0.98	-1.83	-0.12	-0.42	-1.09	0.26
Education level	0.38	-0.06	0.82	-0.19	-0.54	0.16
Physical Frailty	-1.07	-1.54	-0.60	-1.51	-1.89	-1.14
Disability	-1.22	-2.81	0.36	-1.40	-2.65	-0.14
Morbidity	-1.86	-2.86	-0.87	-0.41	-1.20	0.37
Fall History during the Last Year	-0.59	-1.58	0.40	-0.91	-1.69	-0.13
Feeling unhelpful toward family or friends						
Gender	-0.34	-1.35	0.68	-0.26	-1.07	0.54
Age	-0.01	-0.08	0.06	0.06	0.00	0.11
Marital Status	-0.85	-1.70	0.01	-0.35	-1.03	0.33
Education level	0.36	-0.08	0.80	-0.19	-0.54	0.16
Physical Frailty	-1.06	-1.53	-0.59	-1.55	-1.92	-1.18
Disability	-1.08	-2.67	0.50	-1.35	-2.61	-0.09
Morbidity	-1.71	-2.70	-0.72	-0.33	-1.12	0.46
Fall History during the Last Year	-0.55	-1.54	0.44	-0.93	-1.72	-0.14
Not talking to someone every day						
Gender	-0.38	-1.40	0.64	-0.36	-1.16	0.45
Age	-0.03	-0.09	0.04	0.05	0.00	0.10
Marital Status	-0.91	-1.76	-0.05	-0.37	-1.04	0.30
Education level	0.37	-0.07	0.81	-0.21	-0.56	0.14
Physical Frailty	-1.10	-1.57	-0.63	-1.50	-1.88	-1.13
Disability	-1.19	-2.78	0.40	-1.36	-2.61	-0.11
Morbidity	-1.79	-2.79	-0.80	-0.37	-1.15	0.42
Fall History during the Last Year	-0.61	-1.61	0.38	-0.90	-1.68	-0.12
Living alone						
Gender	-0.29	-1.31	0.73	-0.25	-1.05	0.55
Age	-0.02	-0.09	0.04	0.05	0.00	0.11
Marital Status	-0.66	-1.57	0.25	-0.04	-0.75	0.67
Education level	0.40	-0.05	0.84	-0.15	-0.50	0.20
Physical Frailty	-1.19	-1.66	-0.72	-1.59	-1.96	-1.22
Disability	-1.22	-2.82	0.37	-1.41	-2.66	-0.15
Morbidity	-1.79	-2.79	-0.80	-0.15	-1.15	0.42
Fall History during the Last Year	-0.66	-1.65	0.34	-0.96	-1.74	-0.18

Note: B: Unstandardized regression coefficients. CI: 95% Confidence Interval

Bootstrap resample size=5,000

All covariates are controlled in all of the equations for the mediation analyses.

PCS: Physical Component Summary, MCS: Mental Component Summary

Indirect effect: through fear of falling
